# The effectiveness of the WHO school mental health package in promoting mental health literacy among secondary school teachers in Qatar: a randomized controlled trial

**DOI:** 10.1186/s12889-024-19263-6

**Published:** 2024-07-15

**Authors:** Rowaida Elyamani, Omer Nour, Rajvir Singh, Khalid Saeed, Afrah Musa, Noora Alkubaisi, Mohammed Iheb Bougmiza

**Affiliations:** 1 Qatar Metabolic Institute, Hamad Medical Corporation, Doha, 3050 Qatar; 2 Department of Operations, Primary Health Care Corporation, Doha, Qatar; 3 Medical Academic & Research, ACS, Hamad Medical Corporation, Doha, 3050 Qatar; 4 Regional Advisor - Mental Health and Substance Abuse Unit, Department of Non- Communicable Diseases and Mental Health, World Health Organization, Regional Office for the Eastern Mediterranean, Abdul Razzak Al -Sanhouri street, Nasr City, Cairo, 11371 Egypt; 5 Nursing Department, Primary Health Corporation, Doha, Qatar; 6 Clinical Affairs Department, Primary Health Care Corporation, Doha, Qatar; 7 Community Medicine Residency Program - Director, Primary Health Care Corporation, Doha, Qatar; 8 College of Medicine, QU Health, Qatar University, Doha, 2713 Qatar

**Keywords:** Mental health, Intervention, Literacy, Schools, Teachers

## Abstract

Teachers’ mental health literacy (MHL) is fundamental for recognizing and assisting students with mental disorders. The aim of this study was to assess the effectiveness of the World Health Organization School Mental Health Program (WHO-SMHP) in promoting MHL among secondary schools’ teachers in Qatar. We conducted a two-arm randomized control trial. Teachers working in governmental secondary schools were eligible for selection. A total of 16 schools (8 intervention, 8 control) randomly selected and stratified by gender then randomly assigned (1:1). Participants and study team were aware of group assignment. Assessment of teachers’ MHL was at day 0, 3 then 3 months afterward. Bivariat analysis and Generalized Estimating Equations were used in analysis. The primary outcome was teachers MHL. Secondary outcome was the influence of sociodemographic characteristics on the level of MHL. Between October 30, 2018 and March 21, 2019, we randomly assigned 195 teachers from 16 schools to attend training workshop on WHO-SMHP (*n* = 95) or usual practice (*n* = 100). Compared with controls, teachers from intervention group demonstrated a significant improvement in the level of MHL at day three (mean difference = 19.1, 95% CI = 17.0–21.2, Cohen’s d = 2.63; *p* < 0.001).) and after three months (mean difference 16.61, 95% CI = 13.96–19.26, Cohen’s d = 1.86; *p* < 001). There were no significant differences related to influence of other variables on MHL. To our knowledge, this is the first trial to prove the effectiveness of the (WHO-SMHP) in promoting teachers MHL, which can be successfully implemented in a wider scale.

**Supplementary Information:**

The online version contains supplementary material available at 10.1186/s12889-024-19263-6.

## Introduction

Mental health literacy (MHL) is a crucial element for promoting mental health and wellbeing for individuals and populations overall. This is of high importance especially if we aim to overcome barriers of mental health such as lack of knowledge and presence of stigma [1]. MHL was first defined by Jorm AF as “knowledge and beliefs about mental disorders which aid their recognition, management, or prevention” [2]. Mental disorders are life-long conditions that affect general public including adolescents, and interfere with all aspects of their lives. Adolescents however, are at increased risk for many negative outcomes [3]. Studies reported that lack of attention and inappropriate management of mental disorders during adolescence phase, are strong predictors of poor vocational achievements, interpersonal, and family relationship issues [3]. In addition to decreased life expectancy due to associated medical conditions, such as diabetes, heart diseases and stroke, respiratory conditions, and suicide [3, 4].

The World Health Organization reported that more than half of mental disorders starts to manifest during adolescence, and globally one in five adolescents suffers a form of disabling mental disorder, and when they are not taken in consideration, this could result in lifelong adverse consequences, in addition to decrease in compliance with health regimens, and impaired productivity and safety of societies [4]. A systematic review of data from the Eastern Mediterranean Region, showed a raise of more than 10% for the total burden of mental disorders over the past two decades, which outstand the burden in other regions around the globe, including developing regions such as sub-Saharan Africa [5]. Additionally, the review revealed a wide range of the 12-month prevalence of mental illnesses and psychological distresses, between 11.0% and 40.1%, and 15.6% and 35.5%, respectively [5].

These alarming figures have led to global initiation of efforts by international agencies such as the World Health Organization (WHO) and the United Nations Educational, Scientific, and Cultural Organization (UNESCO) to promote school’s mental health [4]. And over the years, many programs have been designed to deliver preventive mental health services in schools, where the majority of adolescents worldwide spend great part of their days. Hence, schools are considered an ideal place to implement activities focused on mental health promotion, prevention and intervention [6]. Teachers, on the other hand, are at the forefront of the universal effort to promote mental health literacy in schools, starting with students in their classrooms. However, researchers found that teachers often lack the knowledge that enables them to recognize students with mental disorders, and encourage professional help seeking for them [7, 8].

In 2016, WHO Eastern Mediterranean Regional Office (WHO EMRO) developed the School Mental Health Package (EMRO SMHP), an evidence-based intervention designed for school staff members including teachers and school counselors [9]. This package was developed by a panel of experts in the field of mental health as well as psychiatrists, and revised through series of editions. Topics covered in the training workshop for this intervention included: Normal childhood development, behavior management strategies for schools, life skills training, essential components for healthy schools, recognizing warning signs for common psychiatric illnesses and developmental disorders and school-based interventions for various psychiatric problems. This intervention, however, has not yet been implemented widely in the region [9]. Murphy J and his team identified and evaluated school-based programs that have been implemented over the past years reaching millions of students in several countries [10]. These large-scale trials have presented some promising findings on the effectiveness of such programs in promoting mental health and helping students who are at risk of psychological dysfunctions. Most of these programs were conducted in high-income countries including mainly the US, but encouraging studies were in progress in countries with limited resources in the South American Continent [10].

Yamaguchi and his colleagues conducted a systematic review to analyze interventional studies on implemented programs, that were designed specifically to enhance MHL among schools’ teachers [11]. Results showed significant improvement separately for knowledge, stigma, and confidence to help students; which are the main three attributes for MHL [11]. Yet, when combining all three, the outcomes reported weak evidence. This systematic review also revealed several limitations in the available programs globally, and the inconsistency of the tools used to assess the level of MHL [11]. Additionally, the lack of validity and reliability of available tools in measuring and assessing MHL is considered a major constraint for future cross-cultural studies, and researchers need to work vigorously on this barrier [11]. By improving teachers’ MHL in schools; they would be able to play a more effective role in the identification, assessment, referral, for mental disorders among schoolchildren and provide an appropriate support for them. This will eventually contribute to promoting mental health in schools. So far, no previous study has been conducted to tackle the subject of MHL in school settings in the local or Gulf region context. We aimed to study the effectiveness of the WHO school-based intervention in improving MHL among secondary schools’ teachers in Qatar, in a sustainable and effective manner.

## Methods

### Study design and participants

We conducted a two-arm randomized control trial (RCT) study design to determine the effectiveness of the WHO SMHP in improving MHL among secondary school teachers in Qatar. The study included governmental secondary schools across the state of Qatar. The study protocol received ethical approval from the institutional review board (IRB) of the medical research center at Hamad Medical Corporation (HMC), as well as approvals from the Ministry of Education and Higher Education (MOEHE) following a vigorous review by the committee and agreed quality assurance for the code of conduct in interventional studies. All methods were carried out in accordance with relevant guidelines and regulations under the IRB. The experimental protocols were approved by the IRB and MOEHE. Additionally, an informed consent was obtained from all subjects and/or their legal guardian(s).

Screening for schools, randomization and allocation were carried by the principal investigator and the informed consent and assessments were collected by assigned and trained data collectors.

The total number of governmental secondary schools in Qatar in 2018 was 57 (29 boys’ schools and 28 girls’ schools), and the student-teacher’s ratio was 9:1. During the screening of schools, principals of two girls schools and one boys school suggested that the intervention timing should be changed as it was near exams period, hence they declined to response at the time of invitation due to shortage of staff to cover for their colleagues if they planned to participate in either the control or intervention groups. These schools were replaced by 3 other schools, and the response rate was nearly 95%. Participants were eligible if they were working in governmental secondary schools. There were no exclusion criteria. Recruitment began on October 30, 2018 and ended on December 10, 2018. Final data were collected on March 21, 2019.

The principal method of recruitment was via sending emails to the principals and administration of selected schools in each study groups, emails included the purpose of the study and schedule of the training workshop for the intervention group, afterward teachers were selected from each school and confirmed their participation to the administration to clear their schedule during the workshop’s days.

We followed the concert statement in this study.

### Randomization and masking

To select schools, we used simple randomization (1:1) with an automated online system, ensuring that the research team was unable to affect randomization. Afterward teachers were selected randomly from each school by the principle to represent each class, an average of 12 teachers from each school, using Microsoft excel sheet to generate teachers’ codes. Participants completed all the assessments independently and therefore their responses could not be affected by the study team.

### Procedures

The intervention was based on the World Health Organization, Eastern Mediterranean Region (WHO-EMRO) School mental health package manual (SMHP) 2016. The package was extensively studies and developed by a team of experts in the field of school mental health in the middle east region. The manual’s goals include teaching educators about the importance of mental health in schools, familiarizing them with child development stages, providing them with proven behavior management techniques, and encouraging mental health promotion using a comprehensive school approach. It covers topics such as social-emotional development in children, creating mental health-promoting schools, addressing student mental health issues in classrooms (including guidance on when to seek further assistance), and includes case studies for a better understanding of common issues. Additionally, standardized handouts are provided to participants for enhanced learning. A group of eight experienced locally-based psychologists were recruited and trained on the content of the intervention by the principal investigators; who received the training from the WHO EMRO country representative that underwent WHO training of trainers (TOT) back in Jordan. The psychologist presented the lectures and delivered the intervention afterward to the teachers involved in the study. The workshop was prepared in forms of series of lectures, audiovisuals, case studies and sessions for group discussions. Location and schedule of the workshop was communicated to teachers in the intervention groups prior to starting date.

The intervention was delivered to all teachers in group 1 (intervention) in one venue for 6 h from 7:30 a.m. to 1:30 p.m., over 3 days (December 17, 19 and 20, 2018). Teachers in the control group received no intervention and carried their work schedule regularly.

Both groups were matched for gender and were assessed at the same time period for their MHL. three points of assessments were; day 0 for baseline assessment (pre-intervention) = T0, day 3 (end of the workshop) = T1, and three months post intervention = T2. All participants signed the informed consents prior to the beginning of intervention. At each point of assessment questionnaires were collected from teachers in sealed coded envelops and handed to the principal investigator to ensure the blinding at the level of data entry and analysis. The code consisted of 4 components; the first letter of the place of birth, the last letter of their fathers’ name, the year of birth, the last two digits of their mobile numbers. All letters were written in English, and Numbers in Arabic numbering.

### Outcomes

The primary outcome was teachers’ MHL; which combines attributes of knowledge, skills, and attitude relevant to the school mental health. These attributes are explained as follows:

- Knowledge about mental disorders: Ability to correctly identify features of a disorder, a specific disorder or category of disorders.
- Knowledge of risk factors and causes: Knowledge of environmental, social, familial or biological factors that increase the risk of developing a mental illness.
- Skills needed to manage mental disorders: steps and strategies that can be applied to handle students suffering from mental disorders in class.
- Attitudes that promote recognition: Attitudes that impact and lead to more recognition of disorders.

Assessment of primary outcome MHL was done through the self-administered tool developed by the WHO to be used in the EMRO region as part of the implementation plan for the WHO EMRO School Mental Health Package. This tool was newly constructed by experts in the region ensuring the validity and tested in a regional country in Arabic language, with fitting contents to the local context. Regarding the face and content validity, the WHO questionnaire was evaluated locally by a group including; eight psychologists working with governmental schools in Qatar and experts in the area of mental health literacy. Additionally, senior consultants in the field community medicine and mom-communicable diseases, and the national trainer of the WHO-SMHP. These participants assessed difficulty, generality and ambiguity of the items, and they were asked to comment on the were asked to comment regarding the grammar of items, choice of vocabulary, placement of items. All 16 psychologists received a 3 days training on the interventions and the questionnaire and they filled the pre-post survey. A permission to use the tool was obtained from the WHO regional advisor of Mental Health and Substance Abuse unit. The first section has 30 items, each item in the questionnaire describes an area of knowledge, skill, or attitude relevant to the school mental health literacy. For each item, participants were asked to express their agreement or disagreement. Each item was scored as 0 = No, 1 = don’t know and 2 = yes. In the second section of the questionnaire, participants were presented with six stories about students suffering from a different form of mental disorder. At the end of each case study, teachers were asked to respond on 5 questions. Each question was scored as 0 = No, and 1 = yes. These questions consisted of items related to teachers’ skills and ability to implement certain techniques that will help students with mental issues, in addition to other items about stigma, and beliefs about interventions. Stories covered the most common psychological problems among students which were; depression, post traumatic disorder syndrome (PTDS), fear, conduct disorder, attention deficit hyperactivity disorder (ADHD), and anxiety disorders. Since social and cultural beliefs may influence answering questions, the characters in stories included female and male students. The score ranged from 0 to 90 with higher scores indicating better MHL. The prior cut-off of 75th percentile was used to determine the adequacy level for MHL overall.

The secondary outcomes for the study were determining influencing factors on the level of MHL. For these outcomes we used a self-administered structured questionnaire constituted of 20 questions related to socio-demographic characteristics (including; age, nationality, level of education, marital status, number of children, level of education, years of experience in Qatar and outside Qatar, subjects taught by teachers: during the academic year 2018–2019, past medical history (including Hypertension that was defined as any history of diagnosis for the patient with the average of casual systolic blood pressure readings ≥ 140 mmHg and/or diastolic pressure readings ≥ 90 mmHg. And Diabetes Miletus type 2 that was defined persistent hyperglycemia in patients with a history of diagnosis of diabetes using the standard tests like fasting blood sugar, Two-Hour Oral Glucose Tolerance Test, and Glycated Hemoglobin (Hb) A1C), past diagnoses with mental disorders, family history of mental disorder, sources of information of mental health, and previous exposure to training on mental health). This section was developed based on extensive literature review and discussion with experts in the field of mental health. Each questionnaire was coded to ensure anonymity and link the same teacher at all three points of assessments (T0, T1, T2).

### Statistical analysis

The required sample size was calculated using the sample size calculation OpenEpi^®^ software version 3.01. In testing *the null hypothesis at* 95% level of confidence Interval (CI), error rate of 5% with power = 80%. For the proportion of exposed with outcome we used 70% based on two studies that showed and improvement of almost 20% in level of mental health literacy after delivering the interventions to teachers [9, 12], while we used 50% for proportion of unexposed with outcome since no similar studies have been conducted in locally or regionally. Adding a 2.5% non-response rate the total would be 195 teachers. The sample size needed for intervention group would be 95 teachers, and 100 for the control group. From each school between 10 and 15 teachers were selected. Since the ratio of 1:1 was applied first to selected schools as the clustering sample unit, however with selecting teachers we gave a range of 12–14 teachers per participating school from all subjects taught. And the selection was done over two days to get the final list of teachers who will attend the workshop, hence there was a small difference in the total number of subjects in each study arm.

Data were entered and analyzed using the Statistical Package for Social Sciences SPSS^®^ V24.0. Quantitative variables were described as mean and standard deviations whereas; frequency with percentages were calculated for categorical variables. Student t tests (independent and dependent) were applied to compare mean difference in scores of MHL between Intervention and control group at time0 (T0), time1 (T1), and time2 (T2). Whereas; chi-square test was applied to compare percentages of categorical variables. If the conditions for the test application were not verified, we used the Yates Corrected Chi Square test. We tested for improvement of score within each group using test of repeated measures, Analysis of Variance (ANOVA). Correlation analysis was performed for time0, time1 and time2 to see associations among them. Being correlated data, General Estimating Equation (GEE) was applied to see effect of intervention, time and other important covariates on MHL score. The method has the advantage of implicitly accounting for data missing at random. We calculated standardized effect sizes with Cohen’s d, dividing the treatment effect by the shared SD. P value 0.05 (two tailed) was considered for statically significant level. Regarding data normality will be tested using kurtosis and skewness tests. The skewness of the teachers scores was found to be -0.116, indicating that the distribution almost normal and only mildly left-skewed. The kurtosis of the scores was found to be -2.01, indicating that the distribution was more moderately tailed compared to the normal distribution.

## Results

**Fig. 1:**
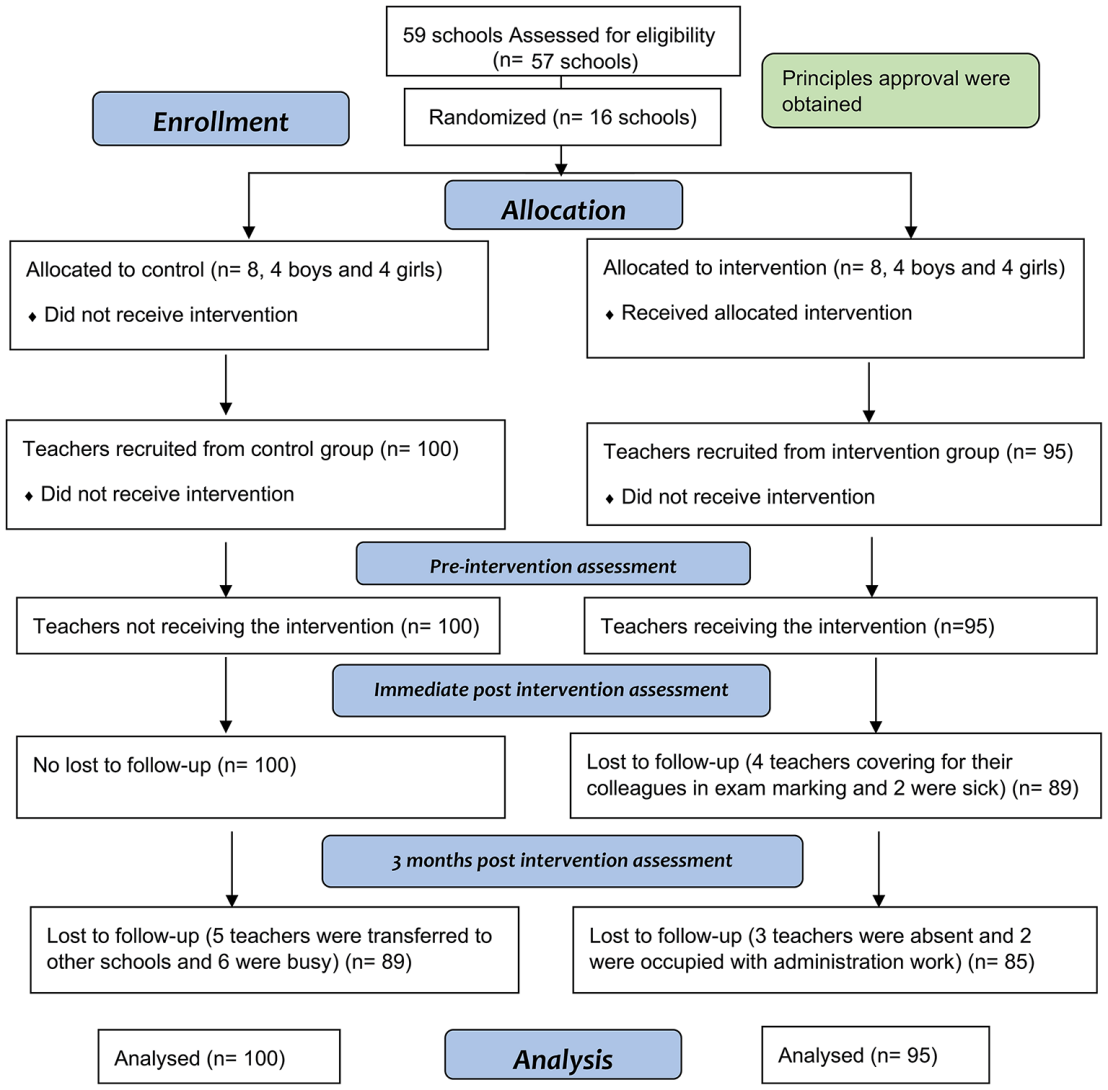
The consort flow diagram. Flow of schools and teachers through the study. All numbers are teachers

Between October 30, 2018, and March 21, 2019, we recruited 195 teachers who were recruited from the randomly assigned 16 secondary schools across the state of Qatar, to receive either the workshop training for three days or no intervention (Fig. 1). Results showed that 49.7% (97/195) of were females and the average age was 41.5 (SD 7.2) years old. The Qatari nationality represented 15% (30/195) of participants. About 70% (135) completed university level of education specially among female, compared to 27% (54/195) of teachers that completed postgraduate degree, mostly for men. The mean for years of experience outside Qatar was 7.2 (SD 6.3) years, and average number of years’ experience inside Qatar 10.1 (SD 9.2) years. A history of chronic diseases was present in 28% (55/195) of participants, mainly Diabetes Mellitus 34.5% (19/55), joint problems 32.7% (18/55) and Hypertension 30.9% (17/55). Less than 9% (16/195) of participants were smoking tobacco at the time of the study, and all of them were males. Almost 3% (5/195) of teachers self-reported being diagnosed with mental illness, and 4% (8/195) had a family member suffering from mental illness. Furthermore, 1 in every 4 (50/195) teachers reported having a form of mental problems, namely anxiety 19% (37/195), stress 15% (30/195) and depression 7% (14/195). The two randomized groups were well matched at baseline (Table 1).

**Table 1:** Characteristics of teachers in study groups (*n* = 195)

Variable	Intervention (*n* = 95) *N* (%)	Control (*n* = 100) *N*(%)
Gender		
Male	47(49.5)	51(51.8)
Mean age (SD)	41(± 7.1)	42(± 7.2)
Marital status		
Single Married Divorced Widowed	10 (10.5) 83 (87.4) 2 (2.1) 0 (0)	5 (5) 94 (94) 0 (0) 1 (1)
Number of children		
0–3 ≥ 4 Missing	61 (64.2) 25 (26.3) 9	53 (53) 31 (31) 16
Level of education		
Secondary University Post graduate degree	4 (4.2) 62 29	2 (2) 73 25
Mean total years of experience (SD)	16.8 (12.5)	17.6 (7.4)
Worked in other jobs	28 (29.5)	20 (20)
Previously diagnosed with mental illness	3 (3.2)	2 (2)
Having a family member diagnosed with mental illness		
No Yes I don’t know	84 1 (1.1) 10 (10.5)	89 7 (7) 4 (4)
Past medical history (yes)	23 (24)	32 (32)
Diabetes mellitus Hypertension Osteoarthritis Others	4 (4.2) 7 (7.4) 7 (7.4) 7 (7.4)	15 (15) 10 (10) 11 (11) 10 (10)
Currently suffering from mental problem	24 (25.3)	26 (26)
Stress Anxiety Depression Others	17 (17.9) 19 (20) 8 (8.4) 2 (2.1)	13 (13.0) 18 (18) 6 (6) 3 (3)
Ever smoked tobacco	11 (11.6)	10 (10)
Looking for information about mental health	85 (89.5)	82 (82)
Previously trained on mental health	25 (26.3)	33 (33)

Data are mean (SD) or n (%)

A total of 189 participants completed the first post intervention assessment (T1) (3.1% dropout), and 174 participants completed the second post intervention assessment (T2) 10.8% dropout). There are 10.5% (10/95) teachers lost to follow-up from the intervention group and 11% (11/100) from the control group at T2 (3 months post assessment). There were no significant differences among dropouts between the two study groups (Table 2). the analyses were conducted per protocol (in the analysis section).

**Table 2:** Comparison of demographic characteristics between the lost to follow-up group and the study participants

Variable	Dropouts *n* = 21 *n* (%)	Study participants *n* = 174 *n* (%)	*p*-value
Gender (female)	11(52.4)	86 (49.4)	0.798
Age mean (SD)	39.6 (5.9)	41.7 (7.3)	0.213
Years of experience mean (SD)	19.91 (20.57)	16.97 (7.55)	0.205
Having history of chronic diseases	4 (19)	51 (29.3)	0.324
Suffering currently from mental problems	5 (23.8)	45 (25.9)	0.839
Previously attended training on mental health	8 (38.1)	50 (28.7)	0.375

The baseline level MHL among teachers of secondary school in Qatar was found to be 25.6% in our study, and this percentage represents those who scored a total above 63 (the 75th percentile). Any score equal to or above 63 was adequate and any score below 63 was labeled inadequate. The intervention group demonstrated a highly significant improvement in their mean scores of MHL at the end of day three of the workshop (mean difference 19.08, 95% CI 17 to 21.16, Cohen’s d = 2.63; *p* < 0.001) and also in the second post intervention assessment after 3 months (mean difference 16.61, 95% CI 13.96 to 19.26, Cohen’s d = 1.86; *p* < 001) (Table 3). We used Cohen’s *d* is in assessing magnitude of effects. Cohen’s *d* was calculated from two mean values of both study groups and their standard deviation (SD). Cohen’s *d* signifies that the mean score of teachers in the intervention group is above the mean score of teachers in the control group, and that the mean score of the intervention group exceeds the scores of 95% percent of those in the control group, when using corresponding percentile to Cohen’s *d.* However, this effect is fading with time but still shows large effect of the workshop on teachers’ level of mental health literacy.

**Table 3:** , difference in mean score of MHL between study arms at T0, T1, T2

Mental Health Literacy tests	Intervention group			Control group			Mean difference	CI	Cohen’s d	*p*-value
	*n*	mean	SD	*n*	mean	SD				
MHL-T0	95	57.6	± 7.6	100	56.5	± 7.1	1.08	-1.01–3.17	0.14	0.31
MHL-T1	89	78.0	± 6.8	100	58.9	± 7.3	19.08	17–21.16	2.63	< 0.001
MHL-T2	85	73.9	± 10.3	89	57.3	± 6.7	16.61	13.96–19.26	1.86	< 0.001

**Fig. 2:**
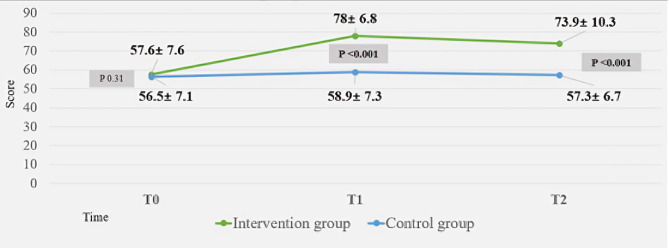
The changes of MHL level in the study groups at T0 (pre-intervention), T1 (immediately after) and T2 (three months post intervention)

The improvement of MHL level in the intervention group from T0 (pre-intervention) to T1 (immediately after) and T2 (three months post intervention) was significant with *p* value of 0.004; the mean score increased from 57.84 at T0 to 73.96 at T2 (Fig. 2).

One-way analysis of variance was used to test significant associations of a number of variables with mental health literacy within teachers in each group separately. variables showed no significant results of MHL mean score within each study group when comparing different categories (Table 4).

**Table 4:** The associations between the level mental health literacy at baseline and different variables for the intervention and control groups

Variable	Intervention			Control		
	Mean (SD)	*p*-value	Effect size	Mean (SD)	*p*-value	Effect size
Gender						
Male Female						
Age						
< 42 ≥ 42	57.8 (7.6) 57.1 (7.8)	0.64	0.002	55.2 (7.2) 57.6 (6.8)	0.09	0.028
Level of education						
Secondary level University level Postgraduate level	60.3 (4.4) 57.7 (7.7) 57.0 (7.9)	0.68	0.008	57.5 (9.1) 56.5 (7.5) 56.5 (5.8)	0.98	< 0.001
Years of experience						
< 16 ≥ 16	57.9 (7.3) 57.3 (8.6)	0.73	0.001	55.6 (7.1) 57.3 (7.0)	0.23	0.014
Past medical history for chronic diseases						
Yes No	57.7 (6.8) 57.5 (7.9)	0.92	< 0.001	57.8 (5.4) 55.9 (7.7)	0.21	0.015
Previous diagnosis with mental illness						
Yes No	60.3 (6.0) 57.5 (7.7)	0.53	0.004	50.5 (9.1) 56.6 (7.0)	0.29	0.015
Suffering from mental problems at the time of the study						
Yes No	58.0 (8.5) 57.4 (7.4)	0.75	0.001	56.8 (8.2) 56.4 (6.7)	0.81	0.001
Looking for information about mental health using different sources						
Yes No	58.3 (6.3) 51.5 (13.9)	0.075	0.007	56.5 (6.8) 65.4 (8.6)	0.95	< 0.001
Previously attended training on mental health						
Yes No	57.2 (7.6) 57.7 (7.7)	0.80	0.001	56.3 (8.6) 56.6 (6.3)	0.84	< 0.001

We performed GEE through having ANOVA followed by regression analysis. Together these tests suggest that MHL score was significantly different between intervention and control group. In other words, analysis of MHL scores revealed a significant time effect (visits at T0, T1 and T2). In addition to visit × group interaction (*p* = 0.001) (Table 5), indicated that the intervention group had significant increase in mental health literacy compared with the control group.

There was strong interaction between visits and group variable. Furthermore, regression analysis showed that the intervention was effective for increasing MHL score in comparison to control group (*p* = 0.001) with visits (*p* = 0.001)as shown in Tables 5 and 6.

**Table 5:** Shows tests of between-subjects effects

Source	Type III Sum of Squares	df	Mean Square	F	Sig.
Corrected Model	62797.382^a^	198	317.158	8.889	0.001
Intercept	2122922.740	1	2122922.740	59497.948	0.001
Group	19402.755	1	19402.755	543.790	0.001
visit	12684.858	2	6342.429	177.756	0.001
visit * Group	8639.264	2	4319.632	121.064	0.001
subj(Group)	21046.814	193	109.051	3.056	0.001

a. r^2^ = 0.831 (Adjusted r^2^ = 0.737)

**Table 6:** Shows important coefficients after regression analysis

Model	Unstandardized Coefficients		Standardized Coefficients		
	B	Std. Error	Beta	T value	*P* value
(Constant)	62.135	1.639		37.910	0.001
Study group	-12.051	0.809	-0.517	-14.889	0.001
visit	4.322	0.489	0.302	8.830	0.001

## Discussion

This study sought to examine the effectiveness of the WHO SMHP intervention in promoting MHL among secondary school teachers in the state of Qatar. Teachers in the intervention group were trained on the WHO SMHP manual for 6 h sessions over three days period. Results showed remarkable improvement in the level of MHL among participants from the intervention group when compared with their counterparts from control group.

Only quarter of secondary schools’ teachers had an adequate level of mental health literacy in the baseline assessment. Similar results were found in a study from Africa where secondary schools teachers had low levels of depression literacy (16.3%), hence, they were unable to identify main symptoms of depression among students [13]. Also, a study in Asia showed that Cambodian teachers struggled to identify common mental disorders among students, which reflects poor mental health literacy, and the most notable areas were relatively poor mental health knowledge, low levels of willingness to interact with people with mental illness, and large proportion of the teachers considered mental disorders as being untreatable [14]. Additionally, research from Europe reported limited levels of MHL among schools’ teachers in the pre-test results, despite the availability of mental health services [15, 16]. In a recent study conducted in four European countries (Hungary, Portugal, Germany and Ireland) on depression literacy among community facilitators; teachers scored very low compared to other professions like nurses [17]. These findings across various scholars may be in part due to the weak role that school mental health programs are imposing in different countries.

Demographic variables in several studies have shown to influence MHL across different population groups, and this included age, gender, years of experiences, chronic illnesses, mental disorders, and resources for mental health information. Despite the numerous studies discussing possible association between MHL and sociodemographic variables, and interaction between them; in this analysis we found that none of those variables influenced the level of MHL among participants, which is somewhat unexpected. This is possibly due to the limited number of experimental studies on MHL among school teachers. Nevertheless, Ganasen et al. argued that among all factors that were linked to MHL, across different scholars, they found that “literacy skills are a stronger predictor of MHL than age, employment status, ethnic group, or educational level” [18]. Farrer and his team showed that older age was associated with increased level of stigma compared to younger generations and justifying this finding with the lack of exposure to media as a readily available source of information among older people [19]. Gender variation in MHL was also evident in number of studies. Considering other related factors like age and cultural context, some scholars reported that females are better in identifying mental disorders compared to males [20, 21]. Some studies revealed that relying on teachers’ experience in dealing with mentally disabled students may be ineffective due to the bias effect of culture believes [22, 23]. Agreeing with these finding, Weston and his colleagues denied any assumptions relating teachers’ experience with better ability in recognition and management of mental problems, since continuous professional training may build new knowledge for all teachers regardless of the length of their career, which they can apply differently [23].

In this trial the most common chronic diseases among teachers were diabetes mellitus, however, the analysis revealed no significant association between MHL and chronic illnesses. The relationship between MHL and chronic illnesses such as hypertension, diabetes, and chronic pain following injuries; are yet to be studied and explored in more depth. Furthermore, Kutcher and his colleague justified in the earliest stages of conceptualizing mental health literacy; that people who have past history of mental disorders or who were in close contact with mentally ill persons may possess a different level of mental health literacy that is influenced by their conditions and their previous attempts to seek professional help [1]. A study from the US among teachers in schools, found that there was minimal training ever provided to them on mental health and common disorders among school students, despite an average year of experience that exceeded a decade, which was reflected negatively in their poor knowledge and weak self-efficacy in handling students with mental issues. Teachers from that same study justified their poor mental health literacy with the insufficient training [24].

Utilizing a randomized controlled study design, this trial was aimed to provide robust evidence of the program across a large representative sample of teachers from multiple high schools across the country. To date, few trials have been conducted to formally test the effectiveness of similar programs within the region, and to our knowledge WHO-SMHP is the first to show actual significant changes in the level of MHL among secondary school teachers over the period of three months, as opposed to several studies that reported little or no significant difference to teachers MHL after introducing interventional programs as found in the latest systematic review by [11]. Similar findings were found in a study by Kutcher et al., where they found significant improvement of teacher’s knowledge about mental disorders, scoring an average of 58.3% (M = 17.5, S.D. = 4.07) at pre-intervention assessment, then reaching up to 76.3% (M = 22.94, S.D. = 2.89), immediately following completion of the training program (*p* < 0.001) [7]. Another study from Tanzania showed a statistically significant improvement in teachers’ knowledge about mental health from average of 14.16 (SD ± 2.19) at the pretraining assessment, to an average of 16.68 (SD ± 2.23) at post-training assessment with (*p* < 0.001) [25]. A Canadian interventional study used the (Guide) curriculum tool to promote teachers MHL in school settings. The intervention was only one session for 8 h, using number of modules, and results showed a significant increase of knowledge in general MHL with the average score of 15 at pre-test and 18 out of 21 questions at post-test, p value of 0.0001 [26].

The duration of MHL interventions also varied greatly in the literature. The WHO-SMHP program lies somewhat in the mid-range of durations reported for interventions on MHL. Some interventions continued over few days [26]. Others for few hours [27]. Only few programs lasted for weeks [28]. Majority of mentioned studies shared a common limitation which is the large numbers of dropouts, this factor may have affected the validity of their results. Additionally, choosing a mid-range program to deliver interventions within the busy schedule of schools’ teachers is not only feasible but may be more appealing to many participants to undertake the training, apply it and benefit from it.

The multiple assessments conducted in the study reflected the possibility of retained knowledge when using the WHO SMHP, which is an advantage over majority of interventional studies in the field of school mental health. Another strength in this trial is being the first study to assess and report promising results using the newly constructed WHO SMHP in the Arabic and local context. We faced a number of limitations in this study, firstly, the study was conducted on governmental schools only and didn’t include private schools which includes almost half of the students and teachers’ populations in the country. Moreover, randomization of schools would have occurred ideally after baseline assessment. However, this was not feasible because schools needed to know in advance whether they were in the intervention or wait list group so that they could schedule their staff training at the start of the school year. Another limitation in this study is the duration of the intervention that is considered long compared to other implemented interventions lasting only day or less, which can leap to more loss of follow up among participants.

## Conclusion

In summary, while majority of teachers showed limited MHL at baseline assessment, participation in the WHO school-based intervention has shown to be effective in improving mental health literacy for secondary school teachers in Qatar compared to no intervention. In doing so, it is likely to equip teachers with the knowledge and skills to not only support their students who suffer mental disorders and challenges, but also their fellow colleagues. These findings highlight the value of providing skills-based programs which can be successfully implemented in a wider scale.

### Electronic supplementary material

Below is the link to the electronic supplementary material.

Supplementary Material 1

## Data Availability

The dataset(s) supporting the conclusions of this article is(are) included within the article (and its additional file(s)).”
